# Supplementary material for: Text classification to streamline online wildlife trade analyses
Source: PLoS One. 2021 Jul 9;16(7):e0254007. doi: 10.1371/journal.pone.0254007 (PMC8270201; doi:10.1371/journal.pone.0254007)
Supplement: S1 Appendix — (DOCX) [file pone.0254007.s001.docx]

# Appendix S1: Definitions of metrics used

## Confusion matrix derived metrics

We evaluated several commonly used machine learning diagnostic metrics derived from confusion matrix values (Appendix S3): true positives (TP), false negatives (FN), true negatives (TN), and false positives (FP) (Fielding and Bell 1997). *Precision* is the proportion of correctly predicted positives compared to all predicted positives. *Recall* is the proportion of correctly predicted positives compared to all observed positives. The *Negative predictive value* is the proportion of correctly predicted negatives compared to all predicted negatives. Finally, the *Specificity* is the proportion of correctly predicted negatives compared to all observed negatives. Mathematically, the metrics are defined (Fielding and Bell 1997) as follows:

$$Precision =\frac{TP}{TP + FP}$$

$$Recall =\frac{TP}{TP + FN}$$

$$Negative predictive value =\frac{TN}{FN + TN}$$

$$Specificity =\frac{TN}{FP + TN}$$

Further, the *F1 score*, is defined as the harmonic mean of precision and recall, mathematically:

$$F1 score = 2 \cdot\frac{Precision \cdot Recall}{Precision + Recall}$$

## Receiver operating characteristic (ROC) curve

The ROC curve shows the performance of a classification model at varying classification thresholds (Fewcett 2006). The curve plots two metrics: False positive rate (i.e., 1 - *Specificity*) and True positive rate (i.e., *Recall*). For each classification threshold (e.g., from 0.01 to 1.0 by units of 0.01), the false positive rate and true positive rate are plotted (e.g., main text Fig 2). The area under the curve for the ROC curve (ROC AUC) is a measure of the positive predictive ability of the classification model (e.g., the ability to predict true positives versus false positives), where an ROC AUC of 0.5 represents positive predictive ability equivalent to chance and an ROC AUC of 1 represents perfect positive predictive ability.

## Precision-Recall (PR) Curve

Like the ROC curve, the precision-recall (PR) curve also displays the performance of a classification model at varying classification thresholds. However, for the PR curve, the tradeoff between *Precision* and *Recall* is examined (not the True versus False positive rate examined in ROC curves). The PR curve is useful when there are imbalanced class sizes (i.e., far fewer positives than negatives) because it does not consider true positives in its calculation (Sofaer et al. 2018).

## References

Fawcett, T. (2006). An introduction to ROC analysis. *Pattern Recognition Letters*, *27*(8), 861–874. <https://doi.org/10.1016/j.patrec.2005.10.010>

Fielding, A. H., & Bell, J. F. (1997). A review of methods for the assessment of prediction errors in conservation presence/absence models. *Environmental Conservation*, *24*(1), 38–49. <https://doi.org/10.1017/S0376892997000088>

Sofaer, H. R., Hoeting, J. A., & Jarnevich, C. S. (2019). The area under the precision-recall curve as a performance metric for rare binary events. *Methods in Ecology and Evolution*, *10*(4), 565–577. <https://doi.org/10.1111/2041-210X.13140>
