# Supplementary material for: Text classification to streamline online wildlife trade analyses
Source: PLoS One. 2021 Jul 9;16(7):e0254007. doi: 10.1371/journal.pone.0254007 (PMC8270201; doi:10.1371/journal.pone.0254007)
Supplement: S2 Appendix — (DOCX) [file pone.0254007.s002.docx]

# Appendix S2: Table of model metrics

The macro-averaged values (10-fold cross validated) of model performance metrics for each label-classifier combination.

| Classifier | ROC AUC | PR AUC | F1 score | Precision | Recall | NPV | Specificity |  |
| --- | --- | --- | --- | --- | --- | --- | --- | --- |
| **domestic poultry** |  |  |  |  |  |  |  |  |
| Logistic Regression | 0.996 | 0.982 | 0.966 | 0.969 | 0.964 | 0.990 | 0.991 |  |
| Naive Bayes | 0.994 | 0.975 | 0.958 | 0.938 | 0.979 | 0.994 | 0.982 |  |
| Random Forest | 0.997 | 0.986 | 0.959 | 0.969 | 0.950 | 0.986 | 0.991 |  |
| **junk** |  |  |  |  |  |  |  |  |
| Logistic Regression | 0.954 | 0.903 | 0.860 | 0.902 | 0.822 | 0.969 | 0.984 |  |
| Naive Bayes | 0.952 | 0.879 | 0.857 | 0.866 | 0.849 | 0.973 | 0.977 |  |
| Random Forest | 0.960 | 0.914 | 0.866 | 0.931 | 0.810 | 0.967 | 0.989 |  |
| **wanted** |  |  |  |  |  |  |  |  |
| Logistic Regression | 0.981 | 0.886 | 0.815 | 0.878 | 0.764 | 0.986 | 0.993 |  |
| Naive Bayes | 0.939 | 0.579 | 0.614 | 0.641 | 0.600 | 0.976 | 0.978 |  |
| Random Forest | 0.987 | 0.893 | 0.775 | 0.913 | 0.676 | 0.981 | 0.996 |  |
