# Supplementary material for: Text classification to streamline online wildlife trade analyses
Source: PLoS One. 2021 Jul 9;16(7):e0254007. doi: 10.1371/journal.pone.0254007 (PMC8270201; doi:10.1371/journal.pone.0254007)
Supplement: S3 Appendix — (DOCX) [file pone.0254007.s003.docx]

# Appendix S3: Confusion “matrices”

The average number (10-fold cross validated) of true positives, false negatives, false positives, and true negatives for each label-classifier combination. The sample size varies slightly across classifiers because (i) the cross-validation split algorithm varies the training and sample set size slightly between cross-validation split and (ii) we report the average (arithmetic mean) 10-fold cross validated values rounded to a whole number. The sample size varies across labels because each label has varying degrees of missing data.

| **Label** | **Classifier** | **True positive** | **False negative** | **False positive** | **True negative** |
| --- | --- | --- | --- | --- | --- |
| **domestic poultry** | Logistic Regression | 349 | 13 | 11 | 1272 |
|  | Naive Bayes | 354 | 8 | 23 | 1260 |
|  | Random Forest | 343 | 18 | 11 | 1272 |
| **junk** | Logistic Regression | 203 | 44 | 22 | 1378 |
|  | Naive Bayes | 210 | 37 | 33 | 1367 |
|  | Random Forest | 200 | 47 | 15 | 1385 |
| **wanted ads** | Logistic Regression | 60 | 19 | 8 | 1288 |
|  | Naive Bayes | 47 | 32 | 29 | 1268 |
|  | Random Forest | 53 | 26 | 5 | 1291 |
