# Supplementary material for: Text classification to streamline online wildlife trade analyses
Source: PLoS One. 2021 Jul 9;16(7):e0254007. doi: 10.1371/journal.pone.0254007 (PMC8270201; doi:10.1371/journal.pone.0254007)
Supplement: Attachment — Submitted filename: Response to Reviewer.pdf [file pone.0254007.s005.pdf]

Reviewer #1: PLOS ONE Manuscript Number: PONE-D-21-05869R1

Text classification to streamline online wildlife trade analyses

I believe the authors have addressed some of the review comments but not all.

In my previous (first) review I mentioned:

“The main problem (specific) is not clearly stated in this paper. From my reading of this paper I can see that this work is a simple and straightforward text classification task with three already well established text classifier (learners) and with the standard text preprocessing steps (stemming, stop-word removal,…etc.); and with standard bag-of-word (feature encoding is not explained in the paper though). With all these, what is the main contribution of this work? I can see that one of the main contributions of this work is the type of problem (wildlife trading). I can see that there is nothing new (nothing unique) about using ML or text classification in this paper.

I think this point is still not clear to me. In other words: What is the gain of this research in the wild-life trade application? You said “to streamline online wildlife…” what do you mean by ‘to streamline”?

> Addressing the aim of our paper, we stated, “Here, we examine the efficacy of text classification models in predicting the relevance of wildlife trade advertisements on the Internet. By “relevance”, we mean “pertaining to the wildlife trade” – filtering out advertisements for e.g., tanks, cages, food, and retaining only genuine advertisements for wildlife.” (lines 72– 74). We believe this makes it abundantly clear what our research aim is.
>
> Addressing the meaning of the word ‘streamline’. We believe that the context of the word is clear from reading the manuscript (i.e., the quoted lines above). Further, the dictionary definition of the word is “make (an organization or system) more efficient and effective by employing faster or simpler working methods”. Thus, for both reasons, we do not know how we can clarify this further. We welcome more specific feedback.

The revisions done on this paper/version are very small (very limited): only a few lines around line 72 and only few lines around line 120; that is it!

> We acknowledge that our changes were minimal, but this was because little was requested to be changed, and our revisions explicitly covered what was asked. We welcome more specific feedback if the Reviewer believes changes are necessary.
