# Supplementary material for: Text classification to streamline online wildlife trade analyses
Source: PLoS One. 2021 Jul 9;16(7):e0254007. doi: 10.1371/journal.pone.0254007 (PMC8270201; doi:10.1371/journal.pone.0254007)
Supplement: Attachment — Submitted filename: Response to Reviewers.docx [file pone.0254007.s004.docx]

PONE-D-21-05869

Text classification to streamline online wildlife trade analyses

PLOS ONE

ACADEMIC EDITOR:

The reviewers have asked for revisions. Some of the reviewers have questioned the novelty of the paper. There is also concerns about the discussion and the comparisons, that authors need to address. Based on all this, I am recommending major revisions.

Furthermore when submitting the revised paper, please also consider the following points:

1. English language needs proofreading.

2. References should be in proper format.

3. All acronyms must first be defined.

We thank the Academic Editor and Reviewer for the comments and feedback. We are encouraged by the overall positive reviews and constructive comments we received. We feel the review process has helped to make our manuscript more rigorous and applicable to the research community. Please find our responses to the Reviewer’s comment below with blue colored and indented text.

We look forward to receiving your revised manuscript.

Kind regards,

Usman Qamar

Academic Editor

PLOS ONE

Additional Editor Comments (if provided):

Reviewer #1: PLOS ONE

Manuscript Number: PONE-D-21-05869

Text classification to streamline online wildlife trade analyses

** The text is not really specific in explaining what is the problem that you are trying to address:

“Here, we examine if text classification models can predict which Internet listings are relevant to wildlife 72 trade research (for our own specific research purposes; e.g., [12]).”

I think you should be more specific.

We thank the Reviewer for their feedback. We have rephrased this sentence for clarity and specificity. It now reads (lines 72 – 73):

“Here, we examine the efficacy of text classification models in predicting the relevance of wildlife trade advertisements on the Internet.”

We further clarify here the context behind our research objectives in the Introduction (Lines 19 – 24):

“Given that many of these advertisements have an unstructured text format, automated identification of relevant listings has not traditionally been possible, nor attempted [for wildlife trade]. Other scientific disciplines have solved similar problems using machine learning and natural language processing models, such as text classifiers. Here, we test the ability of a suite of text classifiers to extract relevant advertisements from an Australian classifieds website where people can post advertisements of their pet birds (n = 16.5k advertisements).”

** I think the domain (application) is very narrow.

We thank the Reviewer for their feedback. We argue that while the techniques are not entirely new, the application to the field of wildlife trade is novel. Please see below (our response to the last Reviewer comment) for a more detailed response on how we revised the manuscript to reflect this objective.

** The (87 Data collection and data curation) section starting line 87 lacks clarity. You manually labeled the advertisement, what are the labels? Did you label them as ‘wanted’, ‘for sale’, ….etc. or as type/species ….etc. what are the species. So many unknowns.

We clarify how the initial labelling occurred in lines (102 - 109):

“For each listing, we manually labelled the taxa (e.g., species) being traded based on the title, the text, and the pictures provided in the listing. Some listings contained more than one species being traded. We identified the listing to the most specific taxonomic rank as possible (species or subspecies), but occasionally not enough information was provided and the listing was identified to genus, family, order, or class (i.e., bird). We resolved taxa names and obtained upper-level taxonomy using the Global Biodiversity Information Facility database [17]. For each listing, we recorded if the user was requesting a bird species (i.e., a wanted advertisement), except in the case of domestic poultry species (see below). We labelled listings not trading a live bird as ‘junk’ (i.e., bird accessory such as cage or bird food).

And further detail how we convert the original labelling to the specific labels used in the text classification models in lines (122 – 128):

“To test the classification of irrelevant listings (see ‘Text classification models’ below), we applied three separate labels for each listing. The first label was for ‘junk’ listings, where a live bird was not being traded (e.g., bird cage). The second label was for ‘wanted’ listings where a user was requesting a bird species and not selling one. The final label was for taxa that we considered non-target for our purposes (i.e., farm, poultry, or domesticated species). We called this label ‘domestic poultry’ and applied it to listings that were selling birds in the taxonomic orders of Anseriformes (waterfowl) and Galliformes (gamebirds) or trading domestic pigeons (Columba livia domestica).”

**how the word features were encoded? Did you use tfidf? Or what?

We have now clarified how the features were encoded (lines 117 - 119):

“For the text classifier models, we tokenized the text to be unigrams (i.e., one word) and did not consider further n-grams. We encoded the unigrams as count vectors and used these vectors as features for the text classification models.”

** in the table (Appendix S3: Confusion “matrices”) the numbers do not match (total 361 vs 362)? Why?

Logistic Regression 348 13

Naive Bayes 354 8

Random Forest 344 18

We thank the Reviewer for this observation. We have changed the text to explain this mismatch. There are 2 reasons why the numbers do not match across classifiers within the same label. First, the cross validation split algorithm does not always split the data equally across each cross-validation fold. Specifically, the algorithm will add 1 additional (or less) row to the test/training dataset depending on the fold (because the 90/10 split does not result in whole numbers). Second, we report the average number of true positives, etc. in the table, thus there is some minor rounding error. We have now included this explanation in the figure legend (Supporting Information lines 53 – 56):

“The average number (10-fold cross validated) of true positives, false negatives, false positives, and true negatives for each label-classifier combination. The sample size varies slightly across classifiers because (i) the cross-validation split algorithm varies the training and sample set size slightly between cross-validation split and (ii) we report the average (arithmetic mean) 10-fold cross validated values rounded to a whole number. The sample size varies across labels because each label has varying degrees of missing data.”

** Also for label junk, the numbers are wrong 246 vs 247? Did you review the paper before submitting?

See above response.

** Overall, the paper is written fairly well (however, more language revision can improve it even more). Several points are not clear in the paper (some are mentioned above in this review). The main problem (specific problem) is not clearly stated in this paper. From my reading of this paper I can see that this work is a simple and straightforward text classification task with three already well established text classifier (learners) and with the standard text preprocessing steps (stemming, stop-word removal,…etc.); and with standard bag-of-word (feature encoding is not explained in the paper though). With all these, what is the main contribution of this work? I can see that one of the main contributions of this work is the type of problem (wildlife trading, maybe I am note sure.). I can see that there is nothing new (nothing unique) about using ML or text classification in this paper.

We agree with the Reviewer’s assessment that our paper does not contribute to new knowledge about ML or text classification. Our purpose was to demonstrate that text classification can be used for data collected on wildlife trade, something that has thus far not been showed. We clarified and emphasize this at several points in the manuscript, including in the Abstract (lines 19 – 22):

“Given that many wildlife-trade advertisements have an unstructured text format, automated identification of relevant listings has not traditionally been possible, nor attempted. Other scientific disciplines have solved similar problems using machine learning and natural language processing models, such as text classifiers.”

In the Introduction (lines 58 – 70):

“Automated methods of data cleaning such as machine-learning techniques and Natural Language Processing (NLP) tools have potential to streamline the processing of wildlife trade data derived from the Internet [10]. A useful but unexplored application is to predict and extract relevant online listings based on their text, which could save time in manual data processing steps if many irrelevant listings exist in the dataset. In particular, text classification models relate the words associated with a particular label, such as ‘relevant’ or ‘irrelevant’, to predict the label of an unknown data point. A well-known application of text classifiers is filtering spam emails [11]. In this context, a text classification model uses a training dataset of labelled emails (spam or not spam) and trains a model to predict those labels based on their constituent words. The resulting model labels new incoming emails as spam or not. In the context of wildlife-trade data derived from the Internet, text classification models have the potential to identify relevant listings and remove irrelevant listings that do not sell wildlife (i.e., fish tanks, bird cages, food) by using the words in the listings. If shown to be effective, this could save researchers substantial time in the data cleaning process.”

In the Discussion (lines 234 – 236):

“Text classification models are commonplace in other disciplines and industries, which work heavily with text data (e.g., [11]), yet have not been applied to data collected on the wildlife trade occurring on the Internet.”

And in the Title of the manuscript:

“Text classification to streamline online wildlife trade analyses”
